# Investigating Water Movement Within and Near Wells Using Active Point Heating and Fiber Optic Distributed Temperature Sensing

**DOI:** 10.3390/s18041023

**Published:** 2018-03-29

**Authors:** Frank Selker, John S. Selker

**Affiliations:** 1 SelkerMetrics, LLC., 4225 SW Agate Lane, Portland, OR 97239, USA; 2 Department of Biological and Ecological Engineering, Oregon State University, Corvallis, OR 97331, USA

**Keywords:** fiber optic, distributed temperature sensing, aquifer characterization, active temperature sensing, fluid velocity measurement, flow measurement, well measurement

## Abstract

There are few methods to provide high-resolution in-situ characterization of flow in aquifers and reservoirs. We present a method that has the potential to quantify lateral and vertical (magnitude and direction) components of flow with spatial resolution of about one meter and temporal resolution of about one day. A fiber optic distributed temperature sensor is used with a novel heating system. Temperatures before heating may be used to evaluate background geothermal gradient and vertical profile of thermal diffusivity. The innovation presented is the use of variable energy application along the well, in this case concentrated heating at equally-spaced (2 m) localized areas (0.5 m). Relative to uniform warming this offers greater opportunity to estimate water movement, reduces required heating power, and increases practical length that can be heated. Numerical simulations are presented which illustrate expected behaviors. We estimate relative advection rates near the well using the times at which various locations diverge from a heating trajectory expected for pure conduction in the absence of advection. The concept is demonstrated in a grouted 600 m borehole with 300 heated patches, though evidence of vertical water movement was not seen.

## 1. Introduction

Our knowledge of the flow of water and oil through aquifers and reservoirs and within wells is often rudimentary, compromising our ability to predict groundwater and contaminant movement. Geological systems are typically heterogeneous, with flow rates varying by orders of magnitude over length scales of meters, yet instrumentation which can reveal this complexity are sparse. A common approach is to hydraulically isolate, or “packer off,” sections of a borehole (well), where an instrument might be placed to assess lateral flow via dilution of salt, dilution of added heat, or physical measurement of flow (e.g., with a hot-wire or spinning flow meter). Each such measurement takes hours to setup and conduct, placing limitations on the spatial and temporal resolution that is practicable. Further, these methods don’t provide information regarding vertical flow in the aquifer.

Geophysical methods characterize geological settings for aquifers, but do not generally allow measurement of flow without injection of electrically conductive tracers (e.g., ground penetrating radar, electrical resistance tomography, gamma transmission, neutron thermalization). Hydraulic tomography is advancing in characterizing permeability fields, but without macro-scale connectivity, the flow which will occur is generally not well constrained unless the number of measurements is greater than the number of values of total parameters to be estimated, which in the case of aquifers is typically on the order of thousands if heterogeneity is to be represented [1].

We present a new method which potentially offers information about the magnitude and direction of vertical flow, as well as the magnitude of lateral flow (the direction of lateral flow can generally be inferred from regional gradients) at meter-scale resolution [2]. The method provides real-time information, so it can also help elucidate how such flows change over time. To accomplish this goal we employed a new implementation of actively heated distributed temperature sensing (AHDTS: [2,3,4,5,6,7,8,9,10,11,12,13,14,15]).

Passive measurement of temperature in boreholes using distributed temperature sensing (DTS) is a long-standing method of the oil industry to monitor location and magnitude of inflows in oil and gas-producing wells (e.g., [16]). The application of AHDTS to boreholes was pioneered by [17] wherein it was shown that the combination of passive measurement of the thermal profile (to obtain the thermal properties of the rock), and uniform actively heated measurements, could reveal magnitudes of lateral flow including values as high as 17 m/d (personal communication of unpublished data, Freifeld, 2010). Characterization of flux with DTS has been demonstrated in dikes and wetlands using much the same strategy, providing useful analytical results for the interpretation of data from this method [13,14,18,19]. While uniformly heated wires have also been used to measure vertical flows in wells [11], localized heating allows delivering more energy to the heated locations, increasing the expected range of flows that may be investigated, and provides additional information in the pattern of cooling near the heated area, as shown in model results below, which may offer information about flow directions at arbitrary angles in formations around the well.

More recently, downhole DTS measurements have been based on the injection of hot water in wells equipped with DTS to characterize flow between boreholes [10] and vertically within bore holes [11,12,20]. While useful in certain circumstances, these methods had several features we hope to avoid. First, we do not wish to inject or withdraw mass, and second, we prefer to seal the borehole to flow so that we might characterize the flow in the aquifer wherein the presence of the borehole will have minimal influence. Finally, we seek a method which can elucidate flow at hundreds of locations simultaneously over extended periods of time. We present a system to achieve these goals.

## 2. Materials and Methods

**Figure 1:**
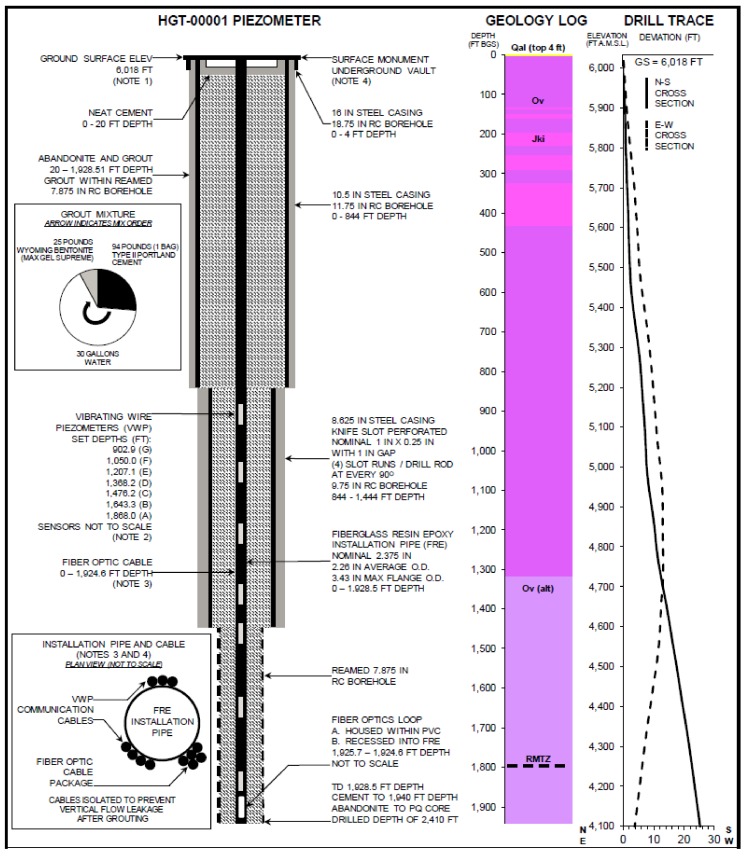
Schematic of borehole showing diameter, piezometers, casing, major geological transitions, and configuration of cables and fiber optic around fiberglass push rod. The geology of the site has three main lithologies: a relatively thin veneer of alluvium (Qal) in the high montane valley overlying the thick, allocthonous Vinini Fm (Ov), which dips northwest. The Vinini also contains Cretaceous intrusions as dikes and veins (Jki) and locally is comprised primarily of diorite. Below around 400 m depth, the siliciclastic Ov contains limy siltstone interbeds where the lithology changes from primarily mudstone/siltstone above to siltstone with greater limestone and minor mudstone below northward trending, underlain by the shallow dipping Roberts Mountain Thrust Zone (RMTZ) which generally acts as a hydraulic barrier [22].

A geotechnical hole which was drilled in early 2011 to characterize the hydrogeology and rock mass in Nevada was made available for this study (for more details see [21] which used the same installation in a passive mode). The borehole was finished as follows: From the surface down to 257 m the 0.3 m diameter borehole was lined with 0.25 m diameter steel casing; from 257 m down to 440 m the 0.25 m borehole was lined with 0.22 m knife-slotted steel casing; from 440 m to the bottom at 591 m the 0.20 m borehole was not cased. Much of the boring down to 411 m (all depths measured from below current ground surface, bgs, noting that about 20 m of material from the site prior to drilling) was broken, low quality very weak rock, and rubble with sand and gravel. A zone of high plasticity clay was observed between 294 and 300 m bgs. From 420 to 556 m bgs was described as more solid and as siltstone rock. However, a section from 455 to 490 m bgs also showed highly fractured and rubble material. Hydraulic conductivity is expected to be lower from 413 to 490 bgs, and the bottom of the upper plate aquifer is estimated to be between 556 and 564 m bgs. The static water level was 122 m bgs. Figure 1 shows a schematic of the borehole, casing, and major geology transitions.

**Figure 2:**
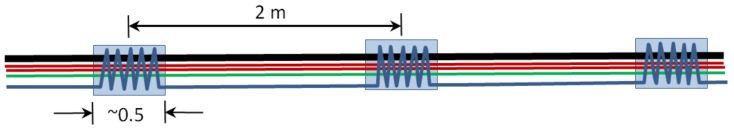
Diagram of typical section of cable. Represented are the main 13.3 mm^2^ copper energy feed cable (black), the 2.08 mm^2^ copper linear heating cable (green, not used), the intermittent, or “dotted line” segmented 5.26 mm^2^ heating cable (blue), and the loop of fiber optic cable (red). The light blue boxes over the wrapped sections represent the HDPE protection sleeves placed over the heating “dots” in the lower 264 m of the installation to prevent abrasion damage during installation.

On 2–3 October 2012 the borehole was fitted with a package of instrumentation, all of which was attached to a 60 mm diameter fiberglass push-tube. The instrumentation included vibrating wire pressure sensors, two heating systems, and a fiber optic temperature sensing cable. The sensing cable consisted of a loop of 3.8 mm O.D. fiber optic sensing cable (BruSense, Brugg, Switzerland) with four bend-tolerant fiber optics (multimode “Clear Curve”, Corning, NY, USA) enclosed in a hydrogen-scavenging gel-filled hermetically-sealed stainless steel tube surrounded by stainless steel strength members enclosed in a nylon jacket (Figure 2). The cable was bent into a U-turn at the bottom in a 20 mm radius 180-degree bend. This bend, together with electrical connections at the borehole bottom, were encased in a 60 mm PVC pipe filled with polyurethane casting compound (SelkerMetrics, Portland, OR, USA). After installation of the instrument the borehole and fiberglass push-tube were filled with bentonite/cement blended grout to seal the well. Warming associated with grout curring dissipated in about 4 weeks, and heating tests reported below were conducted about four months after grouting.

**Figure 3:**
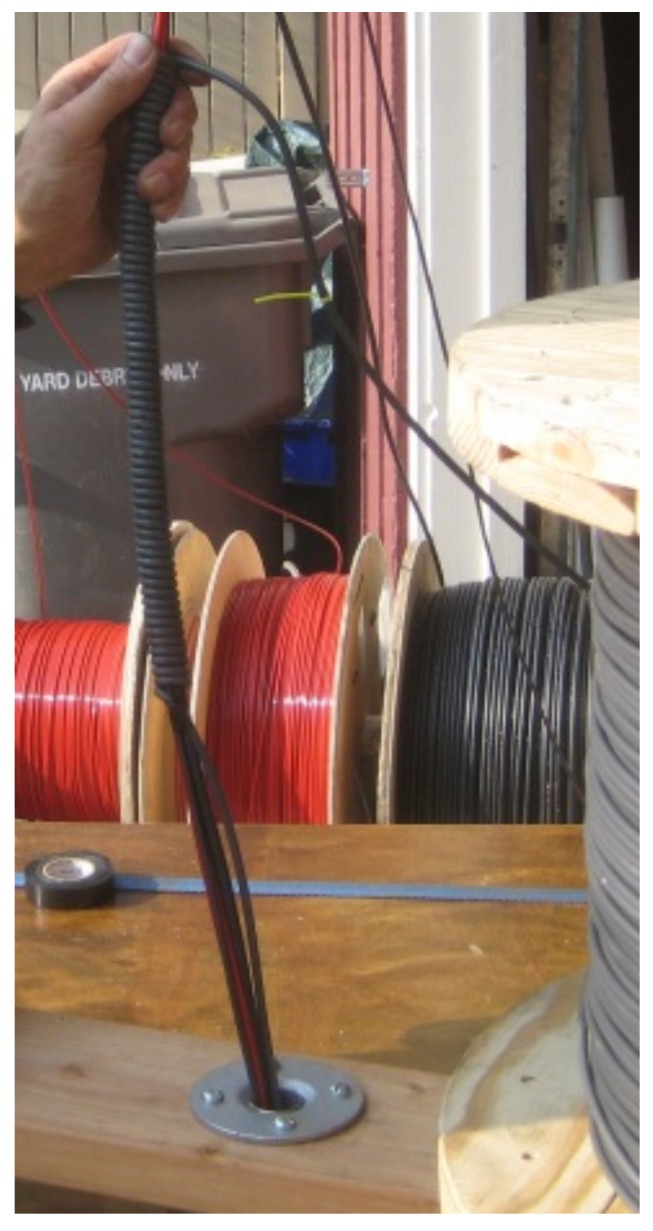
A wrapped section, or heating “dot,” on the cable assembly. In the background the two spools of red fiber optic cable are seen, and to the right, the heavy power feed cable, and to the far right the vertical spool of wrapping cable which was on a rotating holder that allowed wrapping around the other four cables (the linear heating cable spool is not visible).

We constructed a heating package including three heating wires in parallel in the 610 m long cable assembly joined at the bottom of the well (Figure 2 and Figure 3). A thick 610 m copper wire acted as the electrical feed to the well bottom (13.3 mm^2^ of copper providing 0.80 Ohms resistance). A thinner single-strand copper wire (2.08 mm^2^ copper providing 5.0 ohms resistance) was installed for uniform heating, although this was not used for this demonstration. An intermediate thickness wire (5.26 mm^2^ of copper, 1525 m, 5.0 Ohms) was intermittently wrapped about the other cables to create short lengths of high-heating areas (“dots”) separated by lengths with low heating. In the high-heating dots, 6 m of wrapping was applied over a 0.5 m length of the package, providing 12-times more heating per length in the well relative to the sections between dots. To hold the cable assembly in consistent geometry tape bindings were applied at each end of the dots and in two or three locations between successive dots.

During installation the heating cable and vibrating piezometer cables (data not used here) were taped to the fiberglass each meter with 0.05 m wide cloth tape (Figure 1). Abrasion protection (HDPE polyethylene pipe) was applied over the dots on the lower 264 m of cable where there was no well casing to protect from the exposed rock surface. To activate the cable heating system, 240 V was applied between the main feed cable and the dotted-line cable. The uniform heating cable was not activated during this experiment. The current required to energize the dotted line was I = V/R = 240/(5 + 0.8) = 41 A, corresponding to about 10,000 W, or combining the heating of the feed and wrapped cable, an average of 7.9 W/m between dots and when activated 70 W/m within the dots (35 W/dot if taken as a point source, since the dots are 0.5 m in length).

The Distributed Temperature Sensing (DTS) Instrument employed was a Silixa Ultima DTS SR (Silixa, London, UK) which reported temperature every 0.125 m along the cable with a specification of 0.29 m spatial resolution. Temperature measurements made on the down and up passes of the fiber optic cable represented 2300 DTS readings along the installed cable, although only down-pass measurements were used to avoid uncertainty in dot location due to imperfect offset of downward and upward DTS measurement locations. The temperature resolution between adjacent locations is dependent upon the duration of measurements, and with a 15 min integration time giving a resolution of approximately 0.02 °C and resolution during 2 min intervals of approximately 0.05 °C.

The DTS reported about five 12.5 cm elevated temperature locations associated with each dot, so the location of the peak could not be accurately located by simply selecting the highest reported value, since it would not typically occur precisely at the peak. We found that the peak could be located well with a 2-step process involving: (a) interpolating temperatures between reported temperatures to generate a smoother curve, and then (b) fitting a quadratic polynomial to the top three interpolated points and solving for the peak. The method was robust to methods of interpolation, including MatLab’s spline (interp1) or FFT-based smoothing (interpft), and to the coarseness of interpolation smoothing.

Many applications of DTS interpretations are not sensitive to the precise location of measurements, given that reported temperatures are generally the average over between 0.25 and 2.0 m of cable. However, this application demands precisely tracking of the location of temperature peaks associated with each heated dot, and offers the ability to detect instabilities in DTS measurement locations. In analyzing the data we found that the DTS exhibited a roughly ±0.04 m lateral instability over time of reported locations along the cable (see Electronic Supplements 1, Figure S1). Because we could observe a uniform shifting at all peaks as well as in the calibration baths, we could correct for this. We also observed an apparently systematic instrument temperature offset between adjacent temperature measurements, creating alternating low and high readings along the cable (see Electronic Supplements 2, Figure S2). This was corrected by averaging of adjacent values, which should have modest effect on spatial resolution since adjacent measurements are 0.126 m apart but spatial resolution is 0.29 m, or 2.3 times the distance between measures.

Heating of two dots of such an installation under various heating and flow conditions was simulated using the multi-physics finite element modeling system COMSOL 4.3b using the heat transfer modules (COMSOL.com). Here we also use some previously tested analytical results relating observed change in temperature to relative flow velocities in Darcy flow systems for the purposes of demonstration, realizing that given the complexity of the well construction, a numerical inverse solution based on the particular well construction would be needed for estimation of absolute flow velocities, we may however more easily obtain information regarding relative flow velocities. Here we employ the relationship of Fand et al. [23]. Gregory [18] re-arranged Fand et al.’s results to obtain an explicit equation for Darcy flow velocity. For a consistent cable-borehole geometry in a medium of approximately constant thermal properties, the ratio of velocities at any two places can be expressed as:

(1) $$\frac{v_{1}}{v_{2}}={\left[\frac{\Delta T_{2}-C}{\Delta T_{1}-C}\right]}^{2}$$

**Figure 4:**
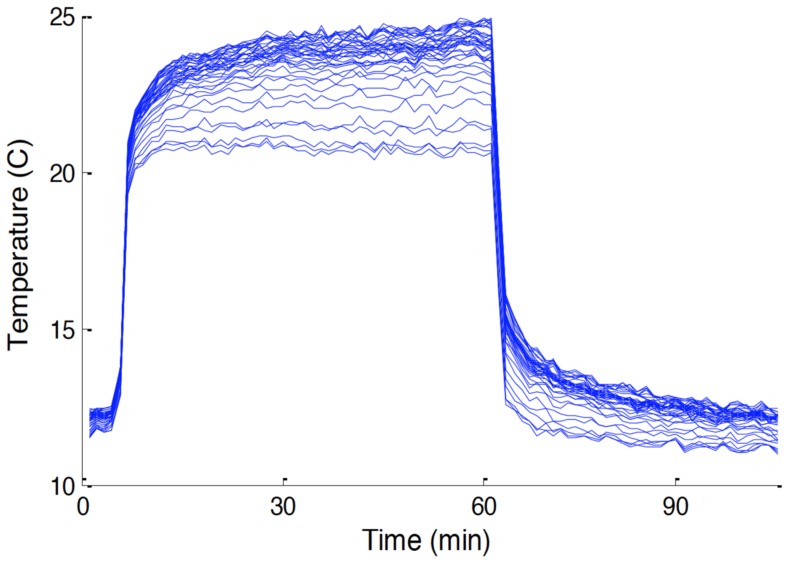
Reproduction of Figure 3.5 in Gregory [18] comparing heating results from 20 section of heated fiber optic cable under an infiltration gallery with spatially variable rates of flow. Locations with low flow continue heating, while those with significant flows plateau.

Note that for locations with significantly different borehole geometries or mediums this estimate of relative velocity will be imprecise, so it is best suited for nearby locations or with supporting information suggesting properties and geometry of compared locations are similar. We see that if we take the *v*_1_ = 0, then Δ*T*_1_ must go to infinity, while if *v*_1_ becomes very large, then Δ*T*_1_ tends to *C*. While temperatures going to infinity might seem unlikely, this would only be with unlimited time, so the observed behavior is simply that no asymptote is achieved. This follows the well-known result of Blackwell [24] who found that for non-moving fluid Δ*T* should be linear with ln(t) with no bound. This behavior was evident in the data of Gregory, shown in Figure 4, where we see both sections that achieve stable values, and those that continue to change temperature after the 1 h of heating employed by Gregory [18].

Perzlmaier et al. [19,25] pointed out that the upper bound on the range of measurement of the heated fiber optics is limited due to the fact that the insulation about the fiber becomes the rate-limiting factor in heat loss at very high flow. This is the source of the C-term in Equation (1). In the formation studied here we know that there are areas of very high flow, and so we estimate C as the value equal to the smallest value of Δ*T* where the temperature is dictated by the insulation quality of the material surrounding the cable rather than the flow velocity. With this simplification we know that velocity will be reported to be off-scale at the location of minimum Δ*T*, and thus we would presume that the top 10% of computed velocity ratios would be highly uncertain, which is the general limitation with this approach (see the same issue explored in detail in the context of heated fiber optic measurement of wind speed found by Sayde et al. [26]).

Another independent approach to estimating flux employs identifying the transition in heating regime from the early diffusion-limited rapid warming to the pseudo-plateau when advection is the dominant loss mechanism. A mathematical framework can be drawn from the analytical solutions of Diao et al. [27] Though they provide the full solution for temperature as a function of the angle of water flow, they show that, regardless of the angle with respect to flow, near the heat source steady temperature will be achieved at time *τ_s_* in a flow field with velocity *u* in a media with density *ρ* and specific heat c (those for water indicated with subscript w) and thermal conductivity *κ*, as described by the remarkably simple equation:

(2) $$u=\sqrt{\frac{4\kappa \rho c}{\tau_{s}\rho^{2}{}_{w}c^{2}{}_{w}}}$$

The time to steady state is a multiple of the cross-over time (denoted *τ_c_*) between early diffusive heating, wherein the temperature change increases proportionally to t^1/2^, and later approaching asymptotically approaching a heating limit determined by advection. So if we define *τ_c_* = E for some constant E, and we care to estimate the relative velocities at two points in the cable that are in media of the same thermal properties we see the constant cancels, and we are left with the simple formula

(3) $$\frac{u_{1}}{u_{2}}=\sqrt{\frac{\tau_{c2}}{\tau_{c1}}}$$

We can gain confidence in this result by obtaining it from an independent approach. Note that the length scale for diffusion in a media with thermal diffusion coefficient of *D* is:

(4) $$L_{diff}=\sqrt{2D\tau }$$

While the length scale for advection is:

(5) $$L_{adv}=u\tau$$

Matching these two length scales (so when diffusion and advection are of equal magnitude in the movement of heat) we find:

(6) $$u=\sqrt{\frac{2D}{\tau }}$$

and so computing the ratio of velocities seen at two locations we again find:

(7) $$\frac{u_{1}}{u_{2}}=\sqrt{\frac{\tau_{c2}}{\tau_{c1}}}$$

This suggests that by computing a time when the data deviates from diffusive heating (e.g., if the difference between fitting Equation 4 to the data exceeds some threshold value of the computed Δ*T*), *τ_c_*, it is straightforward to compute the relative velocities along a heated cable. We will compare the predictions of Equations (1) and (7) to evaluate the consistency of these approaches applied to this data set.

## 3. Results

**Figure 5:**
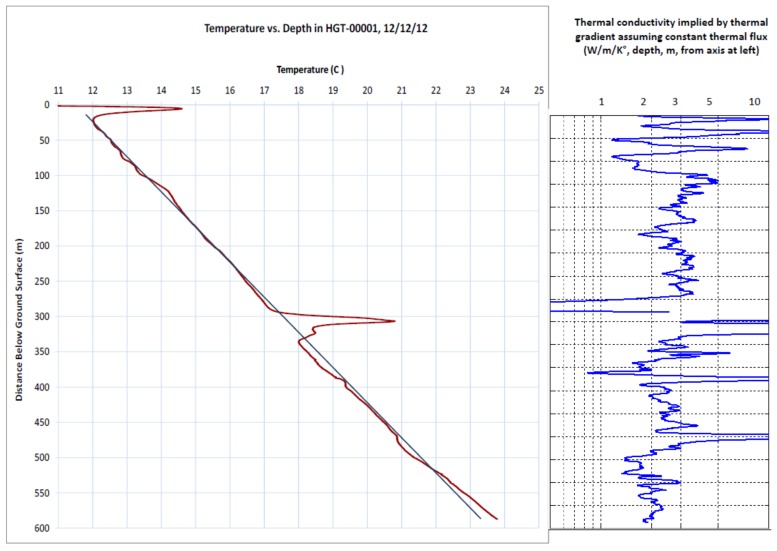
**Left**: Average temperature in borehole with no heating (19:47, 12 December 2012 to 00:02, 13 December 2013). The red line is the measured average temperature and the blue line is a straight line for visual comparison. **Right**: Implied thermal conductivity of formation assuming uniform constant vertical heat flux. Note higher variability above the water table at 122 m and reduced conductivity below about 475 m, where formation is believed to change.

Before heating, the ambient temperature of the bore hole shows typical overall warming with depth due to the vertical geothermal gradient (approximately 0.037 °C/m), with upward deviations near the surface, perhaps due to warming of the rock near the surface due to the exposure of the drilling pad to sun and summer temperatures, as well as a locally warm section at a depth of approximately 300 m believed to be due to lateral flow of drilling fluid from neighboring borehole construction (Figure 5), which is supported by relative advection estimates described below Changes in slopes in the average ambient thermal gradient may be indicative of spatial variation in thermal conductivity of the formation, per the approach of Freifeld et al. [17] but may also reflect localized water movement or heat sources and/or flaws or gaps in grouting, particularly in the knife-slotted and open hole sections.

**Figure 6:**
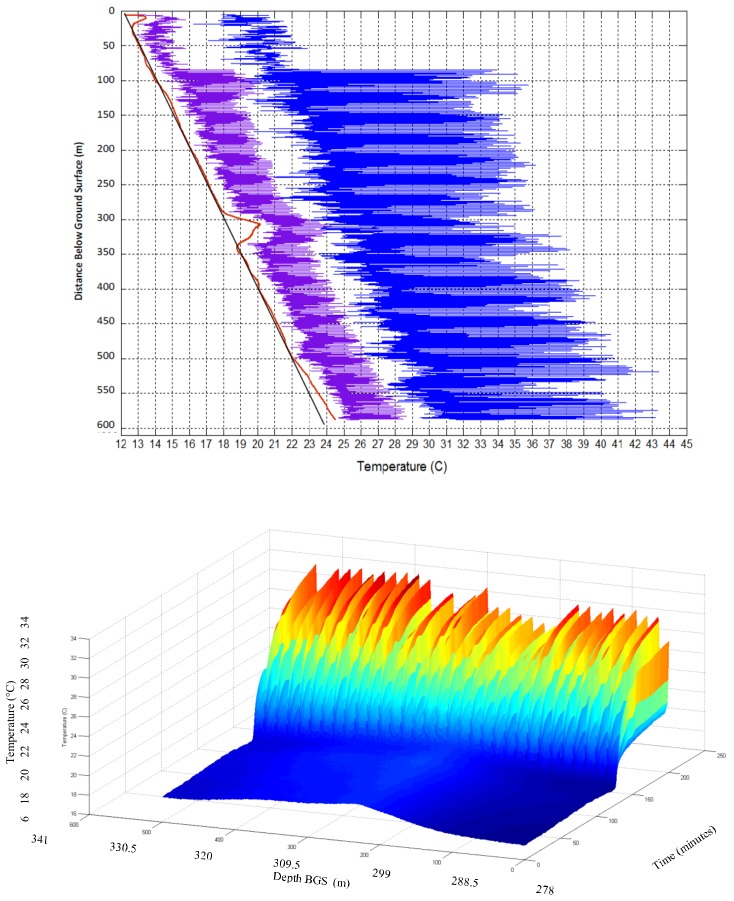
Temperature change after energizing heaters. **Upper**: Temperature prior to the heat pulse (red), 4 min after energizing the intermittent heater (purple), and 24 h after start of heating (blue). **Lower**: “Close-up” view of heating of 50 m portion of sensor. 413 to 490 bgs Brightly colored “fins” that appear with heating are locations of concentrated heat (“dots”). The slightly elevated ridge prior to heating is the anomaly seen in prior to heating from previous drilling activity in the area.

We now consider findings associated with heating. The heating measured after energizing the dotted-line heater was initially rapid—about 1 °C/min during the first 4 min (Figure 6). After 24 h the heated locations (“dots”) had warmed by 15–20 °C, whereas locations between the dots have increased by about 7 °C.

**Figure 7:**
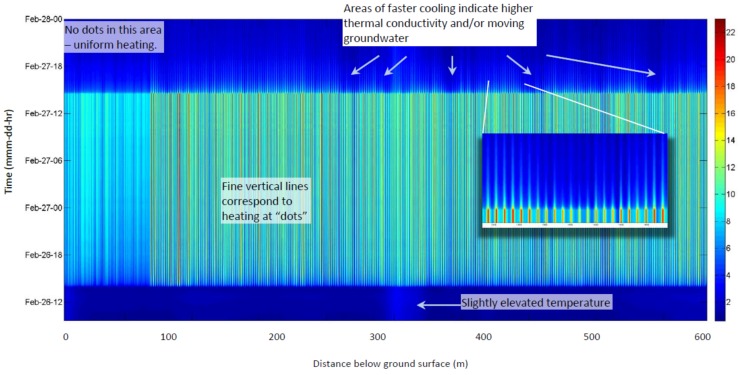
Change in temperature due to energizing dotted-line heater 26–28 February 2012. Time progresses upward, so bottom blue area is pre-heating, vertical lines correspond to locations of concentrated heating (dots); the top blue area is cooling after heating.

Rates of heating and cooling, when energized and then later turned off, can reveal thermal properties of surrounding material and the presence of water movement (advection). The rapid temperature response is followed by a declining rate of increase in temperature for the first eight hours, after which the temperature changes were small as equilibrium of advectively cooled temperature was apparently approached (Figure 4 and Figure 7, more detail below). The decay of temperature mirrors the rise, falling abruptly in the first minutes after power is turned off, then decaying slowly for many hours. Areas of more rapid cooling after the heat is terminated (Figure 7) may correspond to higher thermal conductivity and/or advection due to water movement, as investigated below (e.g., [28]).

**Figure 8:**
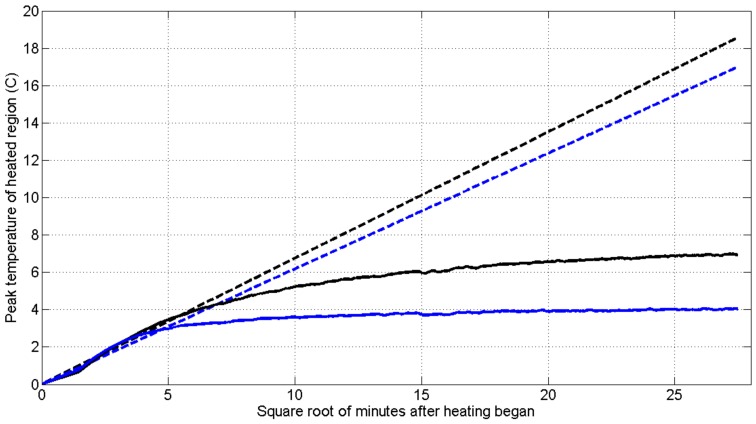
Demonstration for two locations of the diffusion (early time, plots as a straight-line versus square-root of time) versus advection (where the temperature reaches a steady-state where heat loss by advection is balanced by added energy) behavior as introduced in the context of Equations (1) and (2). These data were observed 333 m depth (blue lines) where the recorded temperature (solid line) diverged by 30% from the estimated heating for only conduction (dotted line) after approximately 50 min. At 620 m (black lines) it took approximately 160 min for that degree of divergence to occur, suggesting lower velocity lateral flow.

To further explore how temperature changes distinguish between conduction and advection, Figure 8 shows two heating curves from our installation with differing divergence from heating expected with conduction alone. The temperature expected from pure conduction is extrapolated from the heating that occurs during the first 20 min, during which time conduction is expected to be dominant since the well is fully grouted, preventing fluid movement within the well. Here we base this on taking a typical value of thermal diffusivity for gravel or shale (both of which were seen in the borings, and consistent with the thermal conductivity of 3 W/m·K found in Figure 4) of 8 × 10^−3^ cm^2^/s, and adding about 2 cm^2^/s for the water, gives a value of about 0.01 cm^2^/s [29]. Thus, for the 0.1 m radius well, the approximate thermal diffusive time scale can be computed as t = R^2^/2D = 5000 s = 80 min. Here we are taking the thermal properties of the grout to be about the same as the local rock system, which based on Robertson is not unreasonable. Additionally, differences between grout and rock thermal properties: (a) do not change the expectation of a transition from increasing temperatures if there is pure conduction to steady-state temperatures in the presence of advection, and (b) will affect locations with and without advection similarly. At one location (333 m deep, blue lines) the recorded temperature (solid line) diverges by 30% from the estimated heating if there were only conduction (dotted line) in approximately 50 min. At another location (620 m, black lines) it took approximately 160 min for that same measure of divergence to occur. Using Equation (3) we estimate that relative velocities will be equal to the square ratio of these times, suggesting that flows near the more rapidly diverging location (shallower) are 1.8 times faster than flows past the deeper location.

**Figure 9:**
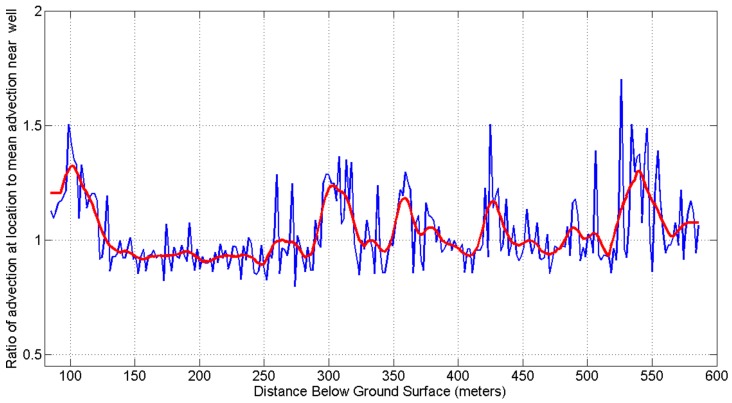
Estimates of relative advective flows for each location along the cable (blue line) from Equation 3, with *τ_C1_* the time for location temperatures to diverge by 30% from temperatures expected with pure conduction, and *τ_C2_* the mean time to diverge by 30%. Smoothing the data (red line) suggests there are several intervals along the well that may have higher rates of advection (e.g., 150–175 m; 370–385 m; and 590–620 m).

Figure 9 shows estimates of relative advective flows for each location along the cable (blue line) from Equation 3, with *τ_C1_* the time for location temperatures to diverge by 30% from temperatures expected with pure conduction, and *τ_C2_* the mean time to diverge by 30%. Values for each location (blue line) are shown together with a moving average (red line, Gausing kernel with standard deviation of 5 min). The results suggest several intervals along the well that may have higher convection rates near the well, including the section showing unusual heating in Figure 6. Other differences in structure may also explain differences, for example differences in thermal properties and fracturing of the surrounding rock, so this method supplements, but does not replace, additional information about a borehole to be investigated. The standard deviation of the residual (original values minus smoothed) values is 0.13. Possible sources of the variability visible in the blue line include variable position of the heater-sensor cable within the well, which may be either against the steel well casing or some distance from the casing within the grout; DTS uncertainty due to the short (2 min) integration times used to capture rapid temperature changes during heating; and algorithm amplification of variable measurements, for example because the modelled conductive heat trajectory is extrapolated from measurements taken over a short period (4 min). The DTS related uncertainty may be reduced by using lower power, so heating occurs more slowly, allowing longer integration times while still capturing the dynamics.

Vertical water movement might be expected to create several possible effects in temperature readings. Perhaps because the borehole was grouted, we saw little evidence of non-lateral water movement, so could not validate this capability in this field installation. However, detecting and potentially quantifying fluid movement within the well over a long distance is a unique capability this sensor may offer, so we will explore expected results of such flow using a finite element model.

**Figure 10:**
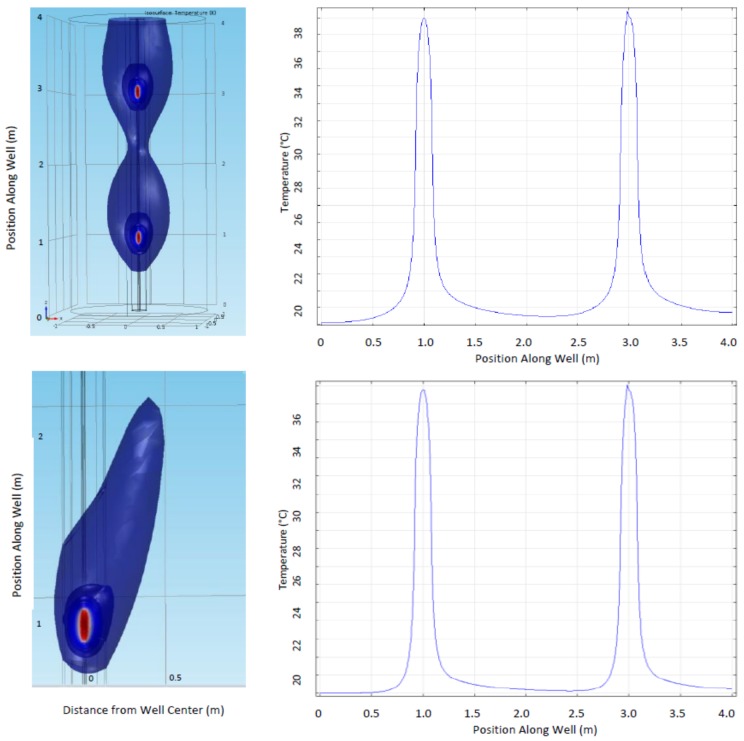
Isothermal surfaces (**left**) and temperature along the central axis of the well (**right**) for water movement (Darcy Flux) of 0.1 m/h in rock surrounding a well modeled using COMSOL 4.3b. Top figures are for vertical, upward flow, bottom is for upward flow at an angle of 15 degrees from vertical. The system is comprised of a heater and DTS sensor centered within 0.1 m of grout within a steel casing, with no water movement within the casing. Temperature along central axis is presented, as would be recorded by DTS, shows asymmetry in peak shoulder shape and cumulative warming as water rises (toward right). Material parameters similar to those expected in the field experiment were used in the modelling, but such a match is approximate since material testing was not conducted within or near the well.

First, there may be asymmetry in the temperature peaks near the dots, as is apparent in numerical simulations of both vertical water movement around the well (upper image and line plot, Figure 10) and for flow that is 15 degrees off of vertical (lower image Figure 10). A second possible effect of vertical water movement would be cumulative heating as the flow warms as it moves past multiple dots. A modest cumulative heating is seen for vertical flow in Figure 10, with slightly increasing temperatures as water moves upward (toward right in line plots). Third, after turning on or off the heat, the peaks could move or change shape if vertically moving water transports heat up or down the DTS. The field data from our demonstration do not conclusively show any of these three effects, perhaps because grouting prevents water movement within the well and nearby vertical movement is insignificant.

## 4. Conclusions

A demonstration installation is presented for a practical method for interrogation of fluid movement in aquifers through the combination of passive observation of temperature, and the response following localized down-hole heating, all observed via DTS. By providing intermittent heat sources in a DTS-monitored borehole, we are able to look for vertical and other non-lateral components of water movement with approximately 2 m resolution shown, and with sub-meter resolution similar to the DTS system being feasible. Temporal resolution was on the order of 2 min, the DTS sampling period. The physical principles underlying the method are founded on well-established concepts in mass and energy transfer in aquifers. We did not observe clear examples of vertical flow in this initial installation, perhaps because the well was grouted and there were not significant vertical flows near the well. We demonstrated the capability to estimate relative advection along the well using the time at which heater-induced warming diverges from the heating trajectory expected for pure conduction in the absence of advection. The complex geological setting, grouting to prevent vertical flow, and absence of corroborating data sources limit the findings. To address these challenges in natural settings we see value in controlled vertical and horizontal flow tests combined with additional numerical simulation to characterize the sensitivity of the method and the capability to quantify flows and thermal conductivities in both the horizontal and vertical directions.

## Supplementary Materials

The following are available online at http://www.mdpi.com/1424-8220/18/4/1023/s1.
