# Supplementary material for: Investigating Water Movement Within and Near Wells Using Active Point Heating and Fiber Optic Distributed Temperature Sensing
Source: Sensors (Basel). 2018 Mar 29;18(4):1023. doi: 10.3390/s18041023 (PMC5948863; doi:10.3390/s18041023)
Supplement: Supplementary file 1 [file sensors-18-01023-s001.pdf]

Investigating Water Movement Within and Near Wells Using Active Point Heating and Fiber Optic Distributed Temperature Sensing

Frank Selker [1,*] and John S. Selker [1,2,*]

[1] SelkerMetrics, LLC., 4225 SW Agate Lane, Portland, OR 97239, USA
[2] Dept. of Biological and Ecological Engineering, Oregon State University, Corvallis, OR 97331, USA *
Correspondence: FSelker@selkermetrics.com, John.Selker@Oregonstate.edu

Electronic Supplement 1

Instrumentation artifact 1: Shifting DTS Sampling Locations Over Time

In looking for subtle movement of heat peaks over time we found evidence that the DTS was slightly changing locations for which temperatures were being reported. We first noticed that all peaks were sometimes moving together in unison. Although unlikely, it was possible that all water in the 600 m well was moving vertically together, so we examined calibration bath data and found the same shift was occurring there, implying that some movement of measurement locations was occurring in the DTS.

Figure S1 shows a measure of the mean depth of heated dots over about 15 h. The variation of about 0.05 m is believed to reflect an artifact of the DTS sampling locations moving slightly over time. A fluctuation of about 0.08 m was observed during the full experiment, which is about two thirds of the distance between adjacent measurements (separated by 0.126 m) and about 27% of the stated spatial resolution of 0.29 m. In some applications such movements would not be important, but in others, such as this application, movement is important to and could affect results. Using calibration bath data this can be corrected for. Ideally there would exist clear and sharp changes in temperature at both ends of the cable that may be monitored for such movement.

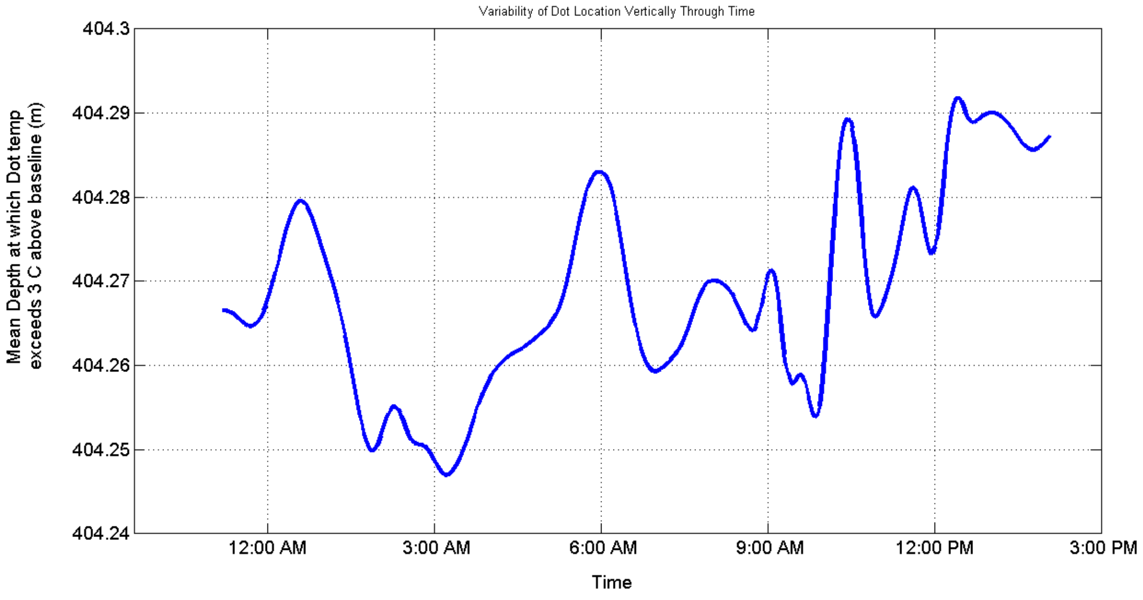

Figure S1. Mean depth of all dots in a 15 h sample. The fluctuation over this interval is about 0.05 m, while total fluctuations of about 0.08 m were observed over the full experiment. The measure of dot location used here is the location at which the heat spike exceeds 3 °C above the baseline temperature. Because the spikes are relatively steep-sided, this measure is believed to accurately capture spike movements. Similar results were found using an alternative method in which the peak of an individual peak was tracked over time.

Electronic Supplement 2

Instrumentation artifact 2: Alternating Temperature Bias at Adjacent DTS Cable Locations

Through the course of the analysis it was observed that temperature measurements along the cable exhibited a small alternating component in adjacent values. In other words, there seemed to be a small alternating positive and negative signal superimposed on the measured signal leading to a "sawtooth" character to the data (Figure S2).

To investigate we calculated the signal's Fourier spectrum and observed a 0.04 spike at the scale of adjacent measurements (red line, far left, Figure S3). We then averaged adjacent temperature measurements and, as expected the spike disappeared (blue line, Figure S3). We used such averaged measurements in the analysis. This does not represent a significant loss in spatial resolution since the DTS unit has a resolution of 0.29 m while adjacent measurements are 0.126 m. Note that in Figure S3 the averaged and raw signals converge at scales of more than about the spatial resolution of about 0.3 m.

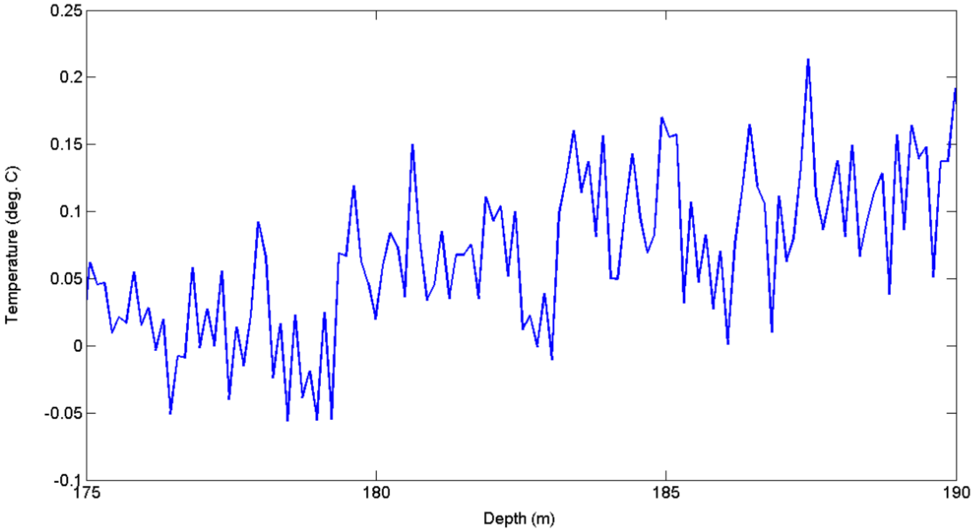

Figure S2. Sample of data suggesting a zig-zag of alternating positive and negative values superimposed on the signal. Temperature sample is from 175 - 190 m in depth taken on 22 April 2013 at 01:44:54. Temperatures are residual values after subtracting a linear fit from data for depth 127.37 m to 256.90 m.

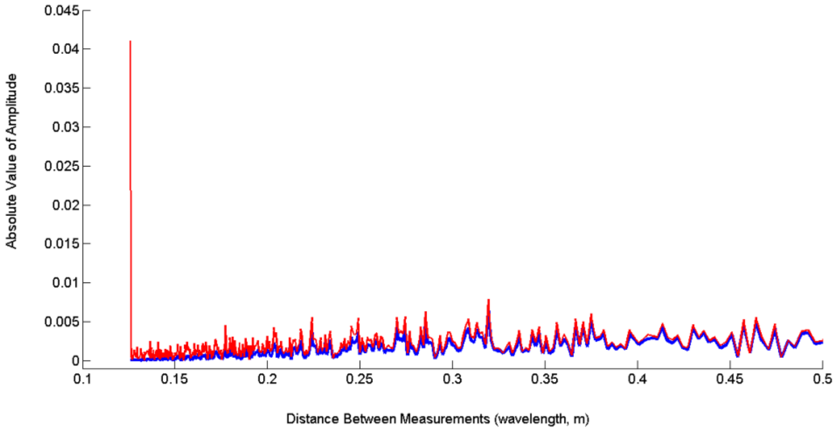

Figure S3. A Fourier analysis was used to visualize the spectrum of a sample of raw data (red). The spike at the shortest interval, between adjacent measurements separated by 0.126 m, shows noise at that frequency corresponding to observed fluctuations in measured temperatures. When the data were smoothed by averaging adjacent readings (blue) this noise disappeared. Such averaging did not affect spatial resolution significantly since the spatial resolution is 0.29 m and the averaging has little impact beyond that scale. The data used for this illustration extended from a depth of 127.37 m to 256.90 m on 22 April 2013 at 01:44:54. Spectra were calculated with MatLab's FFT function after subtracting a linear fit from the data to reduce low-frequency signal elements associated with gradually changing temperatures.
